# Examination of the association of steroids with fluid accumulation in critically ill patients, considering the possibility of biases

**DOI:** 10.1038/s41598-021-85172-y

**Published:** 2021-03-10

**Authors:** Amit Frenkel, Ran Abuhasira, Yoav Bichovsky, Anton Bukhin, Victor Novack, Evgeni Brotfain, Alexander Zlotnik, Moti Klein

**Affiliations:** 1 General Intensive Care Unit, Soroka University Medical Center, and The Faculty of Health Sciences, Ben-Gurion University of the Negev, P.O. Box 151, 84101 Beer-Sheva, Israel; 2 Clinical Research Center, Soroka University Medical Center, and The Faculty of Health Sciences, Ben-Gurion University of the Negev, Beer-Sheva, Israel; 3 The Joyce and Irving Goldman Medical School, Faculty of Health Sciences, Ben-Gurion University of the Negev, Beer-Sheva, Israel; 4 Anesthesia, Critical Care and Pain Medicine, Beth Israel Deaconess Medical Center, Harvard Medical School, Boston, USA; 5 Department of Anesthesiology, Soroka University Medical Center, and The Faculty of Health Sciences, Ben-Gurion University of the Negev, Beer-Sheva, Israel

**Keywords:** Bacterial infection, Diagnosis

## Abstract

Glucocorticoids might have significant influence on positive fluid balance, mostly due to their mineralocorticoid effect. We assessed the association between glucocorticoid therapy and fluid balance in septic patients, in the intensive care unit (ICU). We considered two definitions of exposure: daily exposure to glucocorticoids and glucocorticoid treatment at any time. Of 945 patients, 375 were treated with glucocorticoids in the ICU. We applied four regression models. In the first, fluid balance did not differ during days with and without glucocorticoid treatment, among patients treated and not treated with glucocorticoids in the ICU. In our second model, daily fluid balance was increased in patients who were ever treated with glucocorticoids during their ICU stay compared to untreated patients. In the third model, which included only patients treated with glucocorticoids during their ICU stay, glucocorticoid treatment days were not associated with daily fluid balance. In the last model, on "steroid-free days", patients who received glucocorticoid treatment during their ICU stay had a positive fluid balance compared to those who were never treated with steroids. Despite their known mineralocorticoid activity, glucocorticoids themselves appear not to contribute substantially to fluid retention. This work highlights the importance of precise selection of variables to mitigate biases.

## Introduction

A growing body of evidence suggests that a positive fluid balance in patients hospitalized in intensive care is directly related to worse outcomes^1,2^, and is an independent negative prognostic factor in patients with sepsis^1,3^. Glucocorticoids (GCS) are commonly administered to critically ill patients for a wide range of indications, especially septic shock^4^. GCS might have a substantial influence on a positive fluid balance, mostly due to their mineralocorticoid effect. Increased mineralocorticoid activity and high aldosterone levels cause an increase in sodium reabsorption in the renal tubule^5^. Additionally, some evidence suggests that GCS may overcome the effect of decreased expression of the mineralocorticoid receptor, due to increased levels of tumor necrosis factor-α in critical care patients^6^, thus contributing to fluid retention^7^.

According to the "Surviving Sepsis Campaign" Guidelines for Management of Sepsis and Septic Shock^8^, GCS are recommended for septic shock that is refractory to adequate fluid resuscitation and vasopressor administration. Consequently, patients with sepsis who are treated with GCS are presumably with a more severe disease than patients not treated with GCS. Thus, a possible association between GCS use and fluid balance could be due to the indication for GCS treatment rather than the GCS treatment itself. This raises a core question in the interpretation of observational studies: is the association observed due to a true effect of the exposure or rather the result of a bias?

We conducted a retrospective study to assess the association between GCS therapy and fluid balance in critically ill patients with sepsis. To examine whether a possible association might be due to an indication bias, we analyzed the data according to two definitions of exposure: per GCS treatment at any time during intensive care unit (ICU) stay and GCS treatment per day of stay. In the analysis that used the latter definition, the control group included patients without any GCS treatment and also "steroid-free days" of patients treated with GCS during their ICU stay. We hypothesized that analyses using the two definitions of GCS treatment would yield different results.

## Materials and methods

### Study design and study population

We conducted a population-based retrospective cohort study at Soroka University Medical Center, a tertiary care medical center that serves as the only regional hospital in southern Israel (Beer-Sheva vicinity, estimated population of 1,000,000). We included all adult patients with a diagnosis of sepsis, hospitalized for 24 h or more in the general ICU between December 2006 and January 2018. Sepsis was defined according to either the International Classification of Diseases, 9th revision (ICD-9) codes or a diagnosis in the internal ICU medical record. Comorbidities were also defined by ICD-9 codes. We used the Sequential Organ Failure Assessment (SOFA) score to evaluate organ dysfunction at ICU admission^9^. Exclusion criteria were hemofiltration or dialysis treatment during the ICU stay, and a diagnosis of septic shock that did not appear on the first ICU day.

### Clinical definitions and data sources

GCS treatment was defined as the administration of one of the following drugs, at least once, during the ICU stay: hydrocortisone, methylprednisolone, or prednisone. Indications for steroid treatment were septic shock or acute respiratory distress syndrome. Our center follows the current guidelines for corticoid treatment. During the study period, the indication for such was systolic blood pressure < 90 mmHg for more than one hour following both adequate fluid resuscitation and vasopressor administration (noradrenalin dose > 0.2mcgr/kg/min). Nonetheless, the retrospective study design may have resulted in minor variance in the prescription of steroids.

Using the first definition of GCS exposure, we compared patients who did and did not receive GCS therapy during their ICU stay. Using the second definition of GCS exposure, we aggregated patients' data to 24-h periods. For each of these periods, we analyzed the use of steroids, the daily fluid balance, and serum creatinine for each patient. The daily fluid balance was calculated as the total daily input (nutrition, crystalloids, blood products, intravenous drugs) minus the total daily output (urine, fluids from body drains), and presented in milliliter units. The fluid data is fed automatically into the medical record. We evaluated the creatinine level for every admission day. We used the average daily value when a particular test was performed more than once in a single day. Days in which patients were treated with diuretics were excluded from the analysis. For patients who had more than one admission in the ICU during the study period, we used only the first admission. We limited the analysis to the first 21 ICU hospitalization days. For both definitions of exposure, the outcome was the daily fluid balance during the ICU admission.

### Statistical analysis

The results are presented by means ± SDs for continuous variables, medians and interquartile ranges for ordinal variables, and percentages for categorical data. The Chi-square test was used for categorical variables, the t-test for continuous variables, and the Mann–Whitney test for ordinal variables.

Linear generalized estimating equation (GEE) models with unstructured correlation matrices were used to estimate associations between steroid treatment on each day of admission and the daily fluid balance. GEE models were used to account for repeated measurements of fluid balance in the same patient. In all the models, the dependent variable was the total daily fluid balance in milliliters. The independent variables were defined a priori as fixed effects; firstly, steroid treatment per day or at any time during the ICU stay, depending on the approach used. In addition, the following variables were considered: age, sex, SOFA score at admission, average serum creatinine level, and admission day number in the ICU.

We applied four models to determine the contribution of steroid treatment to the fluid balance (Tables 2, 3,4, 5). In the first model, we compared the fluid balance between all the days with GCS treatment and all the days without GCS. The latter was derived from two sub-groups: the first, "steroid-free days" in patients receiving GCS treatment during their ICU stay; and the second, all ICU days of patients who were not treated with GCS. In the second model, we compared all the days of patients who were not treated with GCS during their ICU stay to all the days of patients who received GCS treatment during their ICU stay. In the third model, we included only the patients who were treated with GCS during their ICU stay. We compared days with GCS treatment to days without GCS treatment. In the last model, we compared all the days of patients who were never treated with GCS during their ICU stay to "steroid-free days" of those who received GCS treatment at some time during their ICU stay. SPSS IBM software, version 25.0, was used for statistical analysis.

### Ethics approval

The study was approved by the Soroka University Medical Center Ethics Committee (EC), reference number 0307–17. All clinical investigations were conducted according to the principles expressed in the Declaration of Helsinki. The EC approval exempted the study from informed consent due to the retrospective data collection that maintained subject confidentiality. Informed consent was waived by Institutional Review Board at "Soroka university medical center" (SUMC). Patient records were anonymized and de-identified prior to analysis.

## Results

### Study population

**Figure 1:**
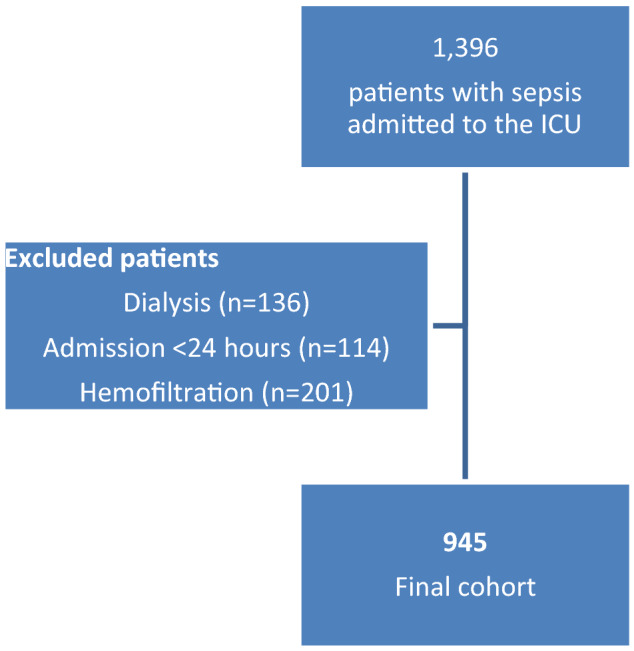
Flow chart – patient selection for this cohort.

The study included 945 patients: 375 (39.7%) who were treated with GCS during their ICU stay and 570 who were not (Fig. 1). The proportions treated by steroids were similar in the early and late years of the study period: 39.3% for 2007–2012 and 40.2% for 2013–2018. Table 1 summarizes the characteristics of the study population: the mean age was lower among those treated than not treated with GCS (57.5 ± 21.3 vs. 61.2 ± 18.9, *p* = 0.006). The majority of the patients in both groups were males (61.6% and 59.5%, respectively). The admission SOFA Score (median, interquartile range (IQR)) was higher among those treated than not treated with GCS (11 (9–13)) vs. (10 (7–12)), *p* < 0.001. Patients with a history of chronic obstructive pulmonary disease constituted 6.1% of those treated with GCS and 1.9% of those not treated (*p* < 0.001). The duration of hospitalization (median, IQR) and the mortality rate in the ICU were higher among those treated than not treated with GCS (10 (3–26) days) vs. (4 (1–14) days) and 25.6% vs. 14.6%, respectively.

**Table 1:** Characteristics of patients at admission to the intensive care unit.

Variable	Treated with steroids during their ICU stay (N = 375)	Not treated with steroids during their ICU stay (N = 570)	*P*-value
Age (Mean ± SD)	61.2 ± 18.9	57.5 ± 21.3	0.006
Male (n, %)	223 (59.5%)	351 (61.6%)	0.52
Comorbidities (n, %)			
CAD	74 (19.7%)	134 (23.5%)	0.17
COPD	23 (6.1%)	11 (1.9%)	0.001
HF	39 (10.4%)	63 (11.1%)	0.75
HTN	189 (50.4%)	263 (46.1%)	0.20
Cancer	89 (23.7%)	103 (18.1%)	0.03
DM	115 (30.7%)	194 (34.0%)	0.28
PVD	20 (5.3%)	42 (7.4%)	0.22
SOFA Score (Median, IQR)	11 (9–13)	10 (7–12)	< 0.001
Cardiovascular sub-score	4 (4–4)	3 (2–4)	< 0.001
Renal sub-score	0 (0–1)	0 (0–1)	< 0.001
Coagulation sub-score	0 (0–1)	0 (0–1)	0.89
Respiration sub-score	3 (2–4)	3 (2–4)	0.003
Liver sub-score	0 (0–1)	0 (0–1)	0.37
CNS sub-score	4 (2–4)	4 (0–4)	0.003
GCS treatment days (Median, IQR)	3 (2–10)	–	–
In-ICU death (n, %)	96 (25.6%)	83 (14.6%)	< 0.001
ICU length of stay	10 (3–26)	4 (1–14)	< 0.001
Mechanically ventilated (n, %)	354 (94.4%)	440 (77.2%)	< 0.001
ARDS	39 (10.4%)	23 (4.0%)	< 0.001
Diuretics treatment days (median, IQR)	3 (1–6)	2 (1–5)	< 0.001

*ICU* intensive care unit, *CAD* coronary artery disease, *COPD* chronic obstructive pulmonary disease, *HF* heart failure, *HTN* hypertension, *DM* diabetes mellitus, *PVD* peripheral vascular disease, *SOFA* sequential organ failure assessment, *GCS* glucocorticoids, *CNS* central nervous system, *ARDS* acute respiratory distress syndrome, *IQR* interquartile range.

### Steroid treatment and fluid balance

**Figure 2:**
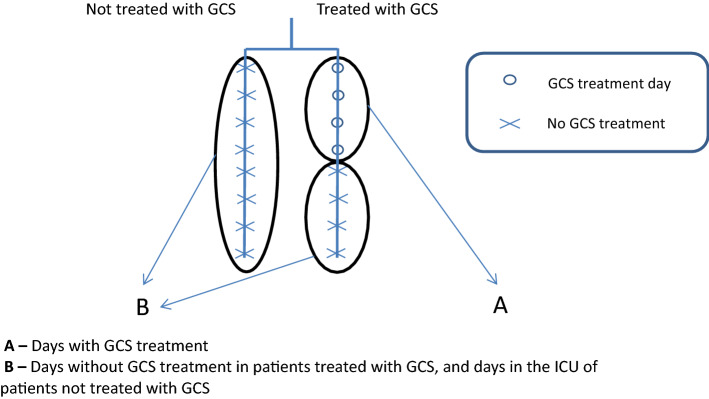
Linked to Table 2 (1st model). The first model compared fluid balance between all the days with GCS treatment and all the days without GCS.

The first model compared fluid balance between all the days with GCS treatment and all the days without GCS. Female sex, older age, higher SOFA score on admission, and high creatinine were all shown to be associated with significantly increased daily fluid balance (Table 2, Fig. 2). This model demonstrated no significant association of GCS treatment with daily fluid balance (coefficient estimate 79.5 (− 55.4 to 214.4), *p*-value = 0.25). Limiting the model to only the first five days of GCS treatment and the first five admission days in patients not treated with GCS yielded similar results.

**Table 2:** Model 1 of the estimated effect of glucocorticoids (GCS) treatment per day on daily fluid balance (ml) in patients with sepsis in the intensive care unit (ICU).

Variable	Coefficient estimate (ml) (95% CI)	*p*-value
Male versus Female	− 291.5 (− 423.9 to − 159.2)	< 0.001
GCS treatment days	79.5 (− 55.4 to 214.4)	0.25
Age (for every added year)	12.1 (9.1 to 15.2)	< 0.001
SOFA admission score (for every added point)	51.9 (30.6 to 73.1)	< 0.001
Creatinine (for every rise in 1 mg/dL)	126.7 (41.7 to 211.7)	0.003

In this model, all the days with GCS were compared to all the days without GCS.

*SOFA* sequential organ failure assessment.

**Figure 3:**
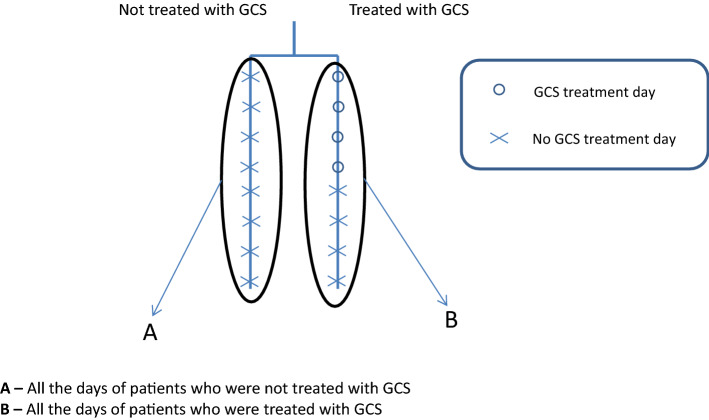
Linked to Table 3 (2nd model). The second model compared all the days hospitalized in the ICU, between patients not treated with GCS and patients who received GCS treatment.

**Figure 4:**
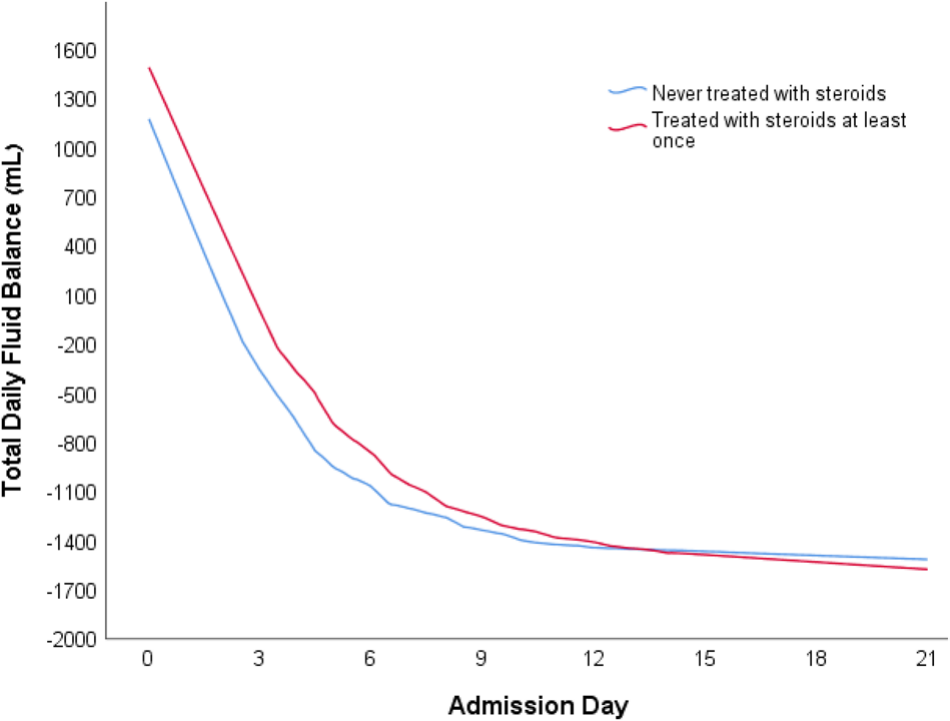
Linked to Table 3 (2nd model). Two locally weighted scatterplot smoothing (LOWESS) curves of the fluid balance in ml throughout the admission days.

The second model compared all the days hospitalized in the ICU, between patients not treated with GCS and patients who received GCS treatment. All the variables mentioned above were associated with significantly increased daily fluid balance (Table 3, Figs. 3, 4). However, the daily fluid balance was increased in patients treated than not treated with GCS, by 139.8 ml (10.8 to 268.9; *p* = 0.03).

**Table 3:** Model 2 of the estimated effect of glucocorticoids (GCS) treatment per day on daily fluid balance (ml) in patients with sepsis in the intensive care unit (ICU).

Variable	Coefficient estimate (ml) (95% CI)	*p*-value
Male versus Female	− 289.7 (− 422.2 to − 157.2)	< 0.001
GCS treatment ever	139.8 (10.8 to 268.9)	0.03
Age (for every added year)	12 (8.9 to 15.1)	< 0.001
SOFA admission score (for every added point)	49.4 (28.2 to 70.5)	< 0.001
Creatinine (for every rise in 1 mg/dL)	129.6 (44.8 to 214.4)	0.003

In this model, all the hospitalized days of patients who were treated with GCS were compared to all the hospitalized days of patients who were not treated with GCS.

*ICU* intensive care unit, *SOFA* sequential organ failure assessment.

*This table is linked to Fig. 4, which presents curves of the fluid balance for the days throughout admission.

**Figure 5:**
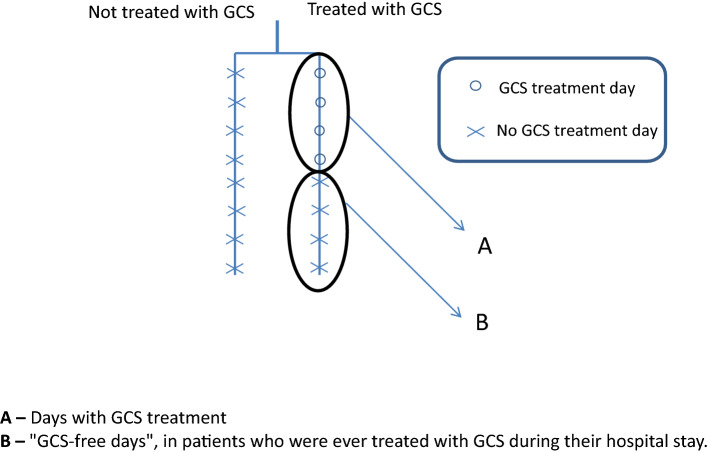
Linked to Table 4 (3rd model). The third model included only patients treated with GCS during their ICU stay and compared "steroid-free days" to days with GCS treatment.

The third model included only patients treated with GCS during their ICU stay and compared "steroid-free days" to days with GCS treatment. We found no significant association of GCS treatment with daily fluid balance (coefficient estimate − 190.6 (− 485.1 to 103.9), *p*-value = 0.21) (Table 4, Fig. 5). This model included 3578 days.

**Table 4:** Model 3 of the estimated effect of glucocorticoids (GCS) treatment per day on daily fluid balance (ml) in patients with sepsis who were treated with GCS during their stay in the intensive care unit (ICU).

Variable	Coefficient estimate (ml) (95% CI)	*p*-value
Male versus Female	− 272.9 (− 476.4 to − 69.3)	0.01
Days with GCS treatment	− 190.6 (− 485.1 to 103.9)	0.21
Age (for every added year)	12.4 (7.3 to 17.6)	< 0.001
SOFA admission score (for every added point)	39.2 (1.7 to 76.7)	0.04
Creatinine (for every rise in 1 mg/dL)	180.4 (41.3 to 319.5)	0.01

This model included only patients treated with GCS during their ICU stay and compared between GCS days and GCS-free days.

*SOFA* sequential organ failure assessment.

**Figure 6:**
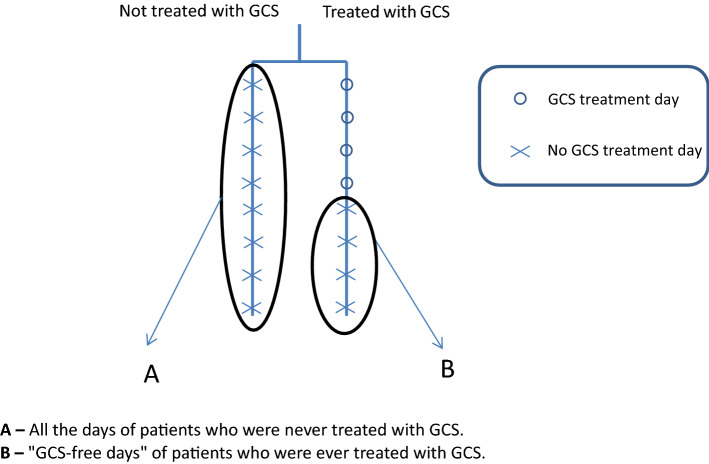
Linked to Table 5 (4rd model). The fourth model compared patients who received GCS treatment during their ICU stay on their "steroid-free days" to those who were never treated with steroids.

In the last model, patients who received GCS treatment during their ICU stay had a positive fluid balance on their "steroid-free days" compared to those who were never treated with steroids (coefficient estimate 157.7 ( − 24.6 to 340.1), *p*-value = 0.09) (Table 5, Fig. 6). All the models, except for the third, included 7775 days.

**Table 5:** Model 4 of the estimated effect of ever being treated with glucocorticoids (GCS) on daily fluid balance (ml) in patients with sepsis in the intensive care unit.

Variable	Coefficient estimate (ml) (95% CI)	*p*-value
Male versus Female	− 250.5 (− 420.2 to − 80.8)	0.004
GCS treatment ever	157.7 (− 24.6 to 340.1)	0.09
Age (for every added year)	12.2 (8 to 16.5)	< 0.001
SOFA admission score (for every added point)	35.5 (5.4 to 65.6)	0.02
Creatinine (for every rise in 1 mg/dL)	100.1 (− 26.4 to 226.7)	0.121

This model included only days without GCS treatment.

*SOFA* sequential organ failure assessment.

## Discussion

Our analysis of data according to two definitions of GCS exposure yielded different results, thus confirming our hypothesis. Specifically, in our cohort of patients with sepsis, we found an association of GCS therapy with positive fluid balance when the exposure was defined as any treatment with GCS during the ICU stay. However, when the exposure was defined as daily exposure to GCS, this association did not hold. Thus, this observational study suggests that evidence of an association between GCS therapy and fluid balance is due to an indication bias, and not to a true effect of GCS treatment. The bias stems from the greater disease severity of the patients treated with GCS. In further support of this interpretation, a positive fluid balance was observed on “steroid-free days” in patients treated with GCS compared to patients not treated with GCS during their ICU stay.

Steroids are widely used in patients with septic shock, while survival benefit has been shown only among those who remained hypotensive after fluid and vasopressor resuscitation^10^. The rationale for glucocorticoid administration in this population is based on data suggesting that critical illness induces a relative adrenal insufficiency that may contribute to shock. Previous studies identified positive fluid balance as a predictor of clinical outcome^2,11^. Moreover, some authors support restrictive intravenous fluid therapy in patients with sepsis to avoid edema within vital organs and organ dysfunction, with impairment of oxygen delivery^11^.

In this study, we applied four models to determine the contribution of steroid treatment to fluid balance in patients hospitalized in the ICU with sepsis. We implemented GEE models to estimate the effect at the population level and to account for repeated measurements. Our first model examined GCS per treatment days. Days with GCS treatment were compared to days without GCS treatment among all the patients in the cohort. This model showed no association of GCS treatment with daily fluid balance. However, in the second model, we compared between patients who received GCS treatment at least once to those who did not receive GCS treatment during their ICU stay. GCS treatment was shown to be associated with a positive daily fluid balance. The conclusion from these two models is that the steroids themselves do not influence the daily fluid balance. Rather, the propensity to treat the more severe patients with GCS may explain the positive balance in those patients. This hypothesis is supported by the third model, which assessed only patients who received GCS treatment. Here, no difference was observed in daily fluid balance between the days with and without GCS treatment. The last model compared only days without GCS treatment, between patients who were and were not treated with GCS. Patients who received GCS treatment at any time during their ICU admission demonstrated positive fluid balance. This again suggests that the clinical characteristics leading to steroid treatment rather than steroids themselves are responsible for the fluid retention. Notably, a remnant effect of GCS treatment may also have contributed to the observed association. The severity of disease in the patients treated with GCS is evidenced by their older age, higher SOFA scores, longer admission times, higher ventilation rates, and higher mortality rates. This is consistent with the indications for GCS according to the guidelines for patients with septic shock^8,10^. The differences in characteristics between the study groups support our supposition that the association between GCS treatment and a positive fluid balance stems from an indication bias, whereby patients with more severe illness and a positive fluid balance are treated with steroids.

The strengths of our study include a lengthened period of data collection with a relatively large cohort. Yet, our study has a number of limitations. First, it is a retrospective study that included patients with sepsis from a single center. Second, we analyzed associations of overall GCS therapy with fluid balance, while the different steroids that were used to treat our patients (hydrocortisone, methylprednisolone) have unequal mineralocorticoid activity^3^. Third, we excluded patients who received renal replacement therapy, due to the impact of such on fluid retention. However, the upshot was the exclusion from the study of a disproportionate number of the more severe patients, who needed renal replacement therapy. Last, though body weight evolution may be a better marker for evaluating fluid balance, our weight data are not accurate enough for such an analysis.

## Conclusions

We suggest that GCS themselves, though known to have mineralocorticoid activity, do not contribute substantially to fluid retention in critically ill patients with sepsis or ARDS. This finding may help elucidate the "fluid balance" concept in critically ill patients with sepsis or ARDS who receive GCS and reduce the concern that GCS causes a positive fluid balance in this population.

## Data Availability

The data used in the analysis of this study are not publicly available due to the national regulations but are available from the corresponding author upon request.
